# Temporal correlation detection using computational phase-change memory

**DOI:** 10.1038/s41467-017-01481-9

**Published:** 2017-10-24

**Authors:** Abu Sebastian, Tomas Tuma, Nikolaos Papandreou, Manuel Le Gallo, Lukas Kull, Thomas Parnell, Evangelos Eleftheriou

**Affiliations:** IBM Research–Zurich, Säumerstrasse 4, 8803 Rüschlikon, Switzerland

## Abstract

Conventional computers based on the von Neumann architecture perform computation by repeatedly transferring data between their physically separated processing and memory units. As computation becomes increasingly data centric and the scalability limits in terms of performance and power are being reached, alternative computing paradigms with collocated computation and storage are actively being sought. A fascinating such approach is that of computational memory where the physics of nanoscale memory devices are used to perform certain computational tasks within the memory unit in a non-von Neumann manner. We present an experimental demonstration using one million phase change memory devices organized to perform a high-level computational primitive by exploiting the crystallization dynamics. Its result is imprinted in the conductance states of the memory devices. The results of using such a computational memory for processing real-world data sets show that this co-existence of computation and storage at the nanometer scale could enable ultra-dense, low-power, and massively-parallel computing systems.

## Abstract (web summary)

New computing paradigms, such as in-memory computing, are expected to overcome the limitations of conventional computing approaches. Sebastian et al. report a large-scale demonstration of computational phase change memory (PCM) by performing high-level computational primitives using one million PCM devices.

## Introduction

**Fig. 1:**
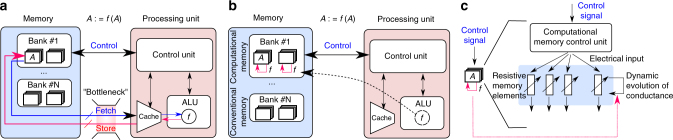
The concept of computational memory. **a** Schematic of the von Neumann computer architecture, where the memory and computing units are physically separated. *A* denotes information stored in a memory location. To perform a computational operation, *f*(*A*), and to store the result in the same memory location, data is shuttled back and forth between the memory and the processing unit. **b** An alternative architecture where *f*(*A*) is performed in place in the same memory location. **c** One way to realize computational memory is by relying on the state dynamics of a large collection of memristive devices. Depending on the operation to be performed, a suitable electrical signal is applied to the memory devices. The conductance of the devices evolves in accordance with the electrical input, and the result of the operation can be retrieved by reading the conductance at an appropriate time instance

In today’s computing systems based on the conventional von Neumann architecture (Fig. 1a), there are distinct memory and processing units. The processing unit comprises the arithmetic and logic unit (ALU), a control unit and a limited amount of cache memory. The memory unit typically comprises dynamic random-access memory (DRAM), where information is stored in the charge state of a capacitor. Performing an operation (such as an arithmetic or logic operation), *f*, over a set of data stored in the memory, *A*, to obtain the result, *f*(*A*), requires a sequence of steps in which the data must be obtained from the memory, transferred to the processing unit, processed, and stored back to the memory. This results in a significant amount of data being moved back and forth between the physically separated memory and processing units. This costs time and energy, and constitutes an inherent bottleneck in performance.

To overcome this, a tantalizing prospect is that of transitioning to a hybrid architecture where certain operations, such as *f*, can be performed at the same physical location as where the data is stored (Fig. 1b). Such a memory unit that facilitates collocated computation is referred to as computational memory. The essential idea is not to treat memory as a passive storage entity, but to exploit the physical attributes of the memory devices to realize computation exactly at the place where the data is stored. One example of computational memory is a recent demonstration of the use of DRAM to perform bulk bit-wise operations^1^ and fast row copying^2^ within the DRAM chip. A new class of emerging nanocale devices, namely, resistive memory or memristive devices with their non-volatile storage capability, is particularly well suited for computational memory. In these devices, information is stored in their resistance/conductance states^3–6^. An early proposal for the use of memristive devices for in-place computing was the realization of certain logical operations using a circuit based on TiO_*x*_-based memory devices^7^. The same memory devices were used simultaneously to store the inputs, perform the logic operation, and store the resulting output. Subsequently, more complex logic units based on this initial concept have been proposed^8–10^. In addition to performing logical operations, resistive memory devices, when arranged in a cross-bar configuration, can be used to perform matrix–vector multiplications in an analog manner. This exploits the multi-level storage capability as well as Ohm’s law and Kirchhoff’s law. Hardware accelerators based on this concept are now becoming an important subject of research^11–17^. However, in these applications, the cross-bar array of resistive memory devices serves as a non-von Neumann computing core and the results of the computation are not necessarily stored in the memory array.

Besides the ability to perform logical operations and matrix–vector multiplications, another tantalizing prospect of computational memory is that of realizing higher-level computational primitives by exploiting the rich dynamic behavior of its constituent devices. The dynamic evolution of the conductance levels of those devices upon application of electrical signals can be used to perform in-place computing. A schematic illustration of this concept is shown in Fig. 1c. Depending on the operation to be performed, a suitable electrical signal is applied to the memory devices. The conductance of the devices evolves in accordance with the electrical input, and the result of the computation is imprinted in the memory array. One early demonstration of this concept was that of finding factors of numbers using phase change memory (PCM) devices, a type of resistive memory devices^18–20^. However, this procedure is rather sensitive to device variabilities and thus experimental demonstrations were confined to a small number of devices. Hence, a large-scale experimental demonstration of a high-level computational primitive that exploits the memristive device dynamics and is robust to device variabilities across an array is still lacking.

In this paper, we present an algorithm to detect temporal correlations between event-based data streams using computational memory. The crystallization dynamics of PCM devices is exploited, and the result of the computation is imprinted in the very same memory devices. We demonstrate the efficacy and robustness of this scheme by presenting a large-scale experimental demonstration using an array of one million PCM devices. We also present applications of this algorithm to process real-world data sets such as weather data.

## Results

### Dynamics of phase change memory devices

**Fig. 2:**
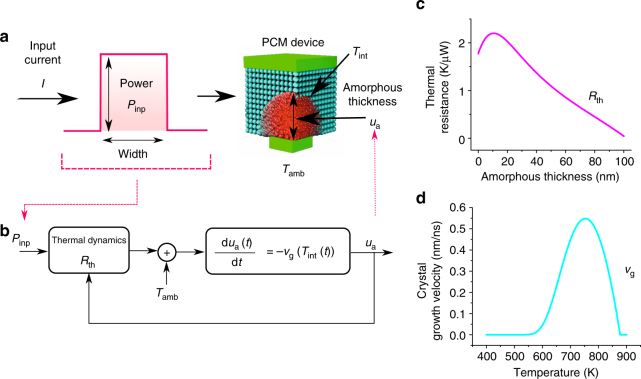
Crystallization dynamics. **a** Schematic of a mushroom-type phase change memory device showing the phase configurations. **b** Illustration of the crystallization dynamics. When an electrical signal with power *P*_inp_ is applied to a PCM device, significant Joule heating occurs. The resulting temperature distribution across the device is determined by the thermal environment, in particular the effective thermal resistance, *R*_th_. The effective thickness of the amorphous region, *u*_*a*_, evolves in accordance with the temperature at the amorphous–crystalline interface, *T*_int_, and with the temperature dependence of crystal growth, *v*_g_. Experimental estimates of **c** *R*_th_ and **d** *v*_g_

A PCM device consists of a nanometric volume of phase change material sandwiched between two electrodes. A schematic illustration of a PCM device with mushroom-type device geometry is shown in Fig. 2a)^21^. In an as-fabricated device, the material is in the crystalline phase. When a current pulse of sufficiently high amplitude is applied to the PCM device (typically referred to as the RESET pulse), a significant portion of the phase change material melts owing to Joule heating. When the pulse is stopped abruptly, the molten material quenches into the amorphous phase because of the glass transition. In the resulting RESET state, the device will be in the low conductance state as the amorphous region blocks the bottom electrode. The size of the amorphous region is captured by the notion of an effective thickness, *u*_a_ that also accounts for the asymmetric device geometry^22^. PCM devices exhibit a rich dynamic behavior with an interplay of electrical, thermal and structural dynamics that forms the basis for their application as computational memory. The electrical transport exhibits a strong field and temperature dependence^23^. Joule heating and the thermal transport pathways ensure that there is a strong temperature gradient within the PCM device. Depending on the temperature in the cell, the phase change material undergoes structural changes, such as phase transitions and structural relaxation^24,25^.

In our demonstration, we focus on a specific aspect of the PCM dynamics: the crystallization dynamics capturing the progressive reduction in the size of the amorphous region due to the phase transition from amorphous to crystalline (Fig. 2b). When a current pulse (typically referred to as the SET pulse) is applied to a PCM device in the RESET state such that the temperature reached in the cell via Joule heating is high enough, but below the melting temperature, a part of the amorphous region crystallizes. At the nanometer scale, the crystallization mechanism is dominated by crystal growth due to the large amorphous–crystalline interface area and the small volume of the amorphous region^24^. The crystallization dynamics in such a PCM device can be approximately described by

1 $$\frac{{{\mathrm{d}}u_{\mathrm{a}}}}{{{\mathrm{d}}t}} = - v_{\mathrm{g}}\left( {T_{{\mathrm{int}}}} \right),$$

where *v*_g_ denotes the temperature-dependent growth velocity of the phase change material; *T*_int_ = *R*_th_(*u*_a_)*P*_inp_ + *T*_amb_ is the temperature at the amorphous–crystalline interface, and $u_{\mathrm{a}}(0) = u_{{\mathrm{a}}_0}$ is the initial effective amorphous thickness^24^. *T*_amb_ is the ambient temperature, and *R*_th_ is the effective thermal resistance that captures the thermal resistance of all possible heat pathways. Experimental estimates of *R*_th_ and *v*_g_ are shown in Figs. 2c, d, respectively^24^. From the estimate of *R*_th_ as a function of *u*_a_, one can infer that the hottest region of the device is slightly above the bottom electrode and that the temperature within the device decreases monotonically with increasing distance from the bottom electrode. The estimate of *v*_g_ shows the strong temperature dependence of the crystal growth rate. Up to approx. 550 K, the crystal growth rate is negligible whereas it is maximum at ~750 K. As a consequence of Eq. 1, *u*_a_ progressively decreases upon the application of repetitive SET pulses, and hence the low-field conductance progressively increases. In subsequent discussions, the RESET and SET pulses will be collectively referred to as write pulses. It is also worth noting that in a circuit-theoretic representation, the PCM device can be viewed as a generic memristor, with *u*_a_ serving as an internal state variable^26–28^.

### Statistical correlation detection using computational memory

In this section, we show how the crystallization dynamics of PCM devices can be exploited to detect statistical correlations between event-based data streams. This can be applied in various fields such as the Internet of Things (IoT), life sciences, networking, social networks, and large scientific experiments. For example, one could generate an event-based data stream based on the presence or absence of a specific word in a collection of tweets. Real-time processing of event-based data streams from dynamic vision sensors is another promising application area^29^. One can also view correlation detection as a key constituent of unsupervised learning where one of the objectives is to find correlated clusters in data streams.

**Fig. 3:**
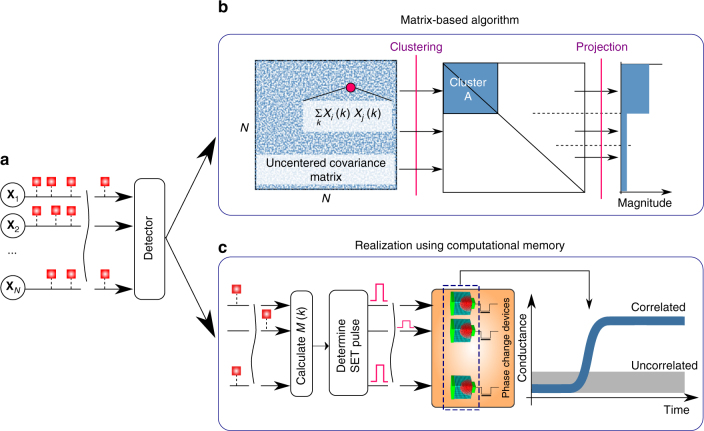
Temporal correlation detection. **a** Schematic of *N* stochastic binary processes, some correlated and the remainder uncorrelated, arriving at a correlation detector. **b** One approach to detect the correlated group is to obtain an uncentered covariance matrix. By summing the elements of this matrix along a row or column, we can obtain some kind of numerical weights corresponding to the *N* processes and can differentiate the correlated from the uncorrelated group based on their magnitudes. **c** Alternatively, the correlation detection problem can be realized using computational memory. Here each process is assigned to a single phase change memory device. Whenever the process takes the value 1, a SET pulse is applied to the PCM device. The amplitude or the width of the SET pulse is chosen to be proportional to the instantaneous sum of all processes. By monitoring the conductance of the memory devices, we can determine the correlated group

In a generic formulation of the problem, let us assume that there are *N* discrete-time binary stochastic processes arriving at a correlation detector (see Fig. 3a). Let *X*_*i*_ = {*X*_*i*_(*k*)} be one of the processes. Then *X*_*i*_(*k*) is a random variable with probabilities

2 $$P\left[ {X_i\left( k \right) = 1} \right] = p$$

3 $$P\left[ {X_i(k) = 0} \right] = 1 - p,$$

for 0 ≤ *p* ≤ 0.5. Let **X**_*j*_ be another discrete-time binary stochastic process with the same value of parameter *p*. Then the correlation coefficient of the random variables *X*_*i*_(*k*) and *X*_*j*_(*k*) at time instant *k* is defined as

4 $$c = \frac{{{\rm Cov}\left[ {X_i(k),X_j(k)} \right]}}{{\sqrt {{\rm Var}\left[ {X_i(k)} \right]{\rm Var}\left[ {X_j(k)} \right]} }}.$$

Processes **X**_*i*_ and **X**_*j*_ are said to be correlated if *c* > 0 and uncorrelated otherwise. The objective of the correlation detection problem is to detect, in an unsupervised manner, an unknown subset of these processes that are mutually correlated.

As shown in Supplementary Note 1 and schematically illustrated in Fig. 3b, one way to solve this problem is by obtaining an estimate of the uncentered covariance matrix corresponding the processes denoted by

5 $$\hat R_{ij} = \frac{1}{K}\mathop {\sum}\limits_{k = 1}^K {X_i(k)X_j(k)} .$$

Next, by summing the elements of this matrix along a row or column, we can obtain certain numerical weights corresponding to the processes denoted by $\hat W_i = \mathop {\sum}\nolimits_{j = 1}^N {\hat R_{ij}}$. It can be shown that if **X**_*i*_ belongs to the correlated group with correlation coefficient *c* > 0, then

6 $$E\left[ {\hat W_i} \right] = \left( {N - 1} \right)p^2 + p + \left( {N_{\mathrm{c}} - 1} \right)cp\left( {1 - p} \right).$$

*N*_c_ denotes the number of processes in the correlated group. In contrast, if **X**_*i*_ belongs to the uncorrelated group, then

7 $$E\left[ {\hat W_i} \right] = \left( {N - 1} \right)p^2 + p.$$

Hence by monitoring $\hat W_i$ in the limit of large *K*, we can determine which processes are correlated with *c* > 0. Moreover, it can be seen that with increasing *c* and *N*_c_, it becomes easier to determine whether a process belongs to a correlated group.

We can show that this rather sophisticated problem of correlation detection can be solved efficiently using a computational memory module comprising PCM devices by exploiting the crystallization dynamics. By assigning each incoming process to a single PCM device, the statistical correlation can be calculated and stored in the very same device as the data passes through the memory. The way it is achieved is depicted schematically in Fig. 3c: At each time instance *k*, a collective momentum, $M(k) = \mathop {\sum}\nolimits_{j = 1}^N {X_j(k)}$, that corresponds to the instantaneous sum of all processes is calculated. The calculation of *M*(*k*) incurs little computational effort as it just counts the number of non-zero events at each time instance. Next, an identical SET pulse is applied potentially in parallel to all the PCM devices for which the assigned binary process has a value of 1. The duration or amplitude of the SET pulse is chosen to be a linear function of *M*(*k*). For example, let the duration of the pulse $\delta t(k) = CM(k) = C\mathop {\sum}\nolimits_{j = 1}^N {X_j(k)}$. For the sake of simplicity, let us assume that the interface temperature, *T*_int_, is independent of the amorphous thickness, *u*_a_. As the pulse amplitude is kept constant, $v_{\rm{g}}{(T_{{\rm{i}} {\rm{n}} {\rm{t}}})} = {\mathscr G}$, where ${\mathscr G}$ is a constant. Then from Eq. 1, the absolute value of the change in the amorphous thickness of the *i* ^th^ phase change device at the *k* ^th^ discrete-time instance is

8 $$\delta u_{{\mathrm{a}}_i}\left( k \right) = \delta t(k)v_{\mathrm{g}}(T_{{\mathrm{int}}}) = C{\mathscr G}\mathop {\sum}\limits_{j = 1}^N X_j(k).$$

The total change in the amorphous thickness after *K* time steps can be shown to be

9 $$\begin{array}{ccccc}\\ \Delta u_{{\mathrm{a}}_i}\left( K \right) = \mathop {\sum}\limits_{k = 1}^K \delta u_{{\mathrm{a}}_i}\left( k \right)X_i\left( k \right)\\ \\ = C{\mathscr G}\mathop {\sum}\limits_{k = 1}^K \mathop {\sum}\limits_{j = 1}^N X_i(k)X_j\left( k \right)\\ \\ = C{\mathscr G}\mathop {\sum}\limits_{j = 1}^N \mathop {\sum}\limits_{k = 1}^K X_i\left( k \right)X_j\left( k \right)\\ \\ = KC{\mathscr G}\mathop {\sum}\limits_{j = 1}^N \hat R_{ij}\\ \\ = KC{\mathscr G}\hat W_i.\\ \end{array}$$

Hence, from Equations 6 and 7, if **X**_*i*_ is one of the correlated processes, then $\Delta u_{{\mathrm{a}}_i}$ will be larger than if **X**_*i*_ is one of the uncorrelated processes. Therefore by monitoring $\Delta u_{{\mathrm{a}}_i}$ or the corresponding resistance/conductance for all phase change devices we can determine which processes are correlated.

### Experimental platform

**Fig. 4:**
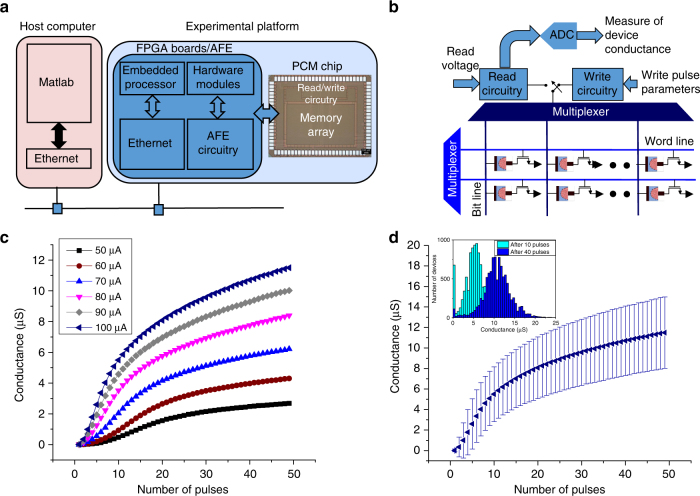
Experimental platform and characterization results. **a** Schematic illustration of the experimental platform showing the main components. **b** The phase change memory array is organized as a matrix of word lines (WL) and bit lines (BL), and the chip also integrates the associated read/write circuitries. **c** The mean accumulation curve of 10,000 devices showing the map between the device conductance and the number of pulses. The devices achieve a higher conductance value with increasing SET current and also with increasing number of pulses. **d** The mean and standard deviation associated with the accumulation curve corresponding to the SET current of 100 μA. Also shown are the distributions of conductance values obtained after application of the 10th and 40th SET pulses

Next, we present experimental demonstrations of the concept. The experimental platform (schematically shown in Fig. 4a) is built around a prototype PCM chip that comprises 3 million PCM devices. More details on the chip are presented in the methods section. As shown in Fig. 4b), the PCM array is organized as a matrix of word lines (WL) and bit lines (BL). In addition to the PCM devices, the prototype chip integrates the circuitry for device addressing and for write and read operations. The PCM chip is interfaced to a hardware platform comprising two field programmable gate array (FPGA) boards and an analog-front-end (AFE) board. The AFE board provides the power supplies as well as the voltage and current reference sources for the PCM chip. The FPGA boards are used to implement the overall system control and data management as well as the interface with the data processing unit. The experimental platform is operated from a host computer, and a Matlab environment is used to coordinate the experiments.

An extensive array-level characterization of the PCM devices was conducted prior to the experimental demonstrations. In one experiment, 10,000 devices were arbitrarily chosen and were first RESET by applying a rectangular current pulse of 1 μs duration and 440 μA amplitude. After RESET, a sequence of SET pulses of 50 ns duration were applied to all devices, and the resulting device conductance values were monitored after the application of each pulse. The map between the device conductance and the number of pulses is referred to as accumulation curve. The accumulation curves corresponding to different SET currents are shown in Fig. 4c. These results clearly show that the mean conductance increases monotonically with increasing SET current (in the range from 50 and 100 μA) and with increasing number of SET pulses. From Fig. 4d, it can also be seen that a significant variability is associated with the evolution of the device conductance values. This variability arises from inter-device as well as intra-device variability. The intra-device variability is traced to the differences in the atomic configurations of the amorphous phase created via the melt-quench process after each RESET operation^30,31^. Besides the variability arising from the crystallization process, additional fluctuations in conductance also arise from 1/*f* noise^32^ and drift variability^33^.

### Experimental demonstration with a million processes

**Fig. 5:**
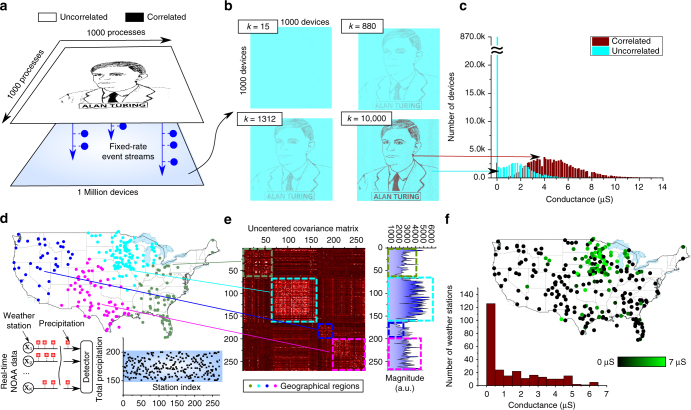
Experimental results. **a** A million processes are mapped to the pixels of a 1000 × 1000 pixel black-and-white sketch of Alan Turing. The pixels turn on and off in accordance with the instantaneous binary values of the processes. **b** Evolution of device conductance over time, showing that the devices corresponding to the correlated processes go to a high conductance state. **c** The distribution of the device conductance shows that the algorithm is able to pick out most of the correlated processes. **d** Generation of a binary stochastic process based on the rainfall data from 270 weather stations across the USA. **e** The uncentered covariance matrix reveals several small correlated groups, along with a predominant correlated group. **f** The map of the device conductance levels after the experiment shows that the devices corresponding to the predominant correlated group have achieved a higher conductance value

In a first demonstration of correlation detection, we created the input data artificially, and generated one million binary stochastic processes organized in a two-dimensional grid (Fig. 5a). We arbitrarily chose a subset of 95,525 processes, which we mutually correlated with a relatively weak instantaneous correlation coefficient of 0.1, whereas the other 904,475 were uncorrelated. The objective was to see if we can detect these correlated processes using the computational memory approach. Each stochastic process was assigned to a single PCM device. First, all devices were RESET by applying a current pulse of 1 μs duration and 440 μA amplitude. In this experiment, we chose to modulate the SET current while maintaining a constant pulse duration of 50 ns. At each time instance, the SET current is chosen to be equal to $0.002 ^ \ast M(k)$ μA, where $M(k) = \mathop {\sum}\nolimits_{j = 1}^N X_j(k)$ is equal to the collective momentum. This rather simple calculation was performed in the host computer. Alternatively, it could be done in one of the FPGA boards. Next, the on-chip write circuitry was instructed to apply a SET pulse with the calculated SET current to all PCM devices for which **X**_*i*_(*k*) = 1. To minimize the execution time, we chose not to program the devices if the SET current was less than 25 μA. The SET pulses were applied sequentially. However, if the chip has multiple write circuitries that can operate in parallel, then it is also possible to apply the SET pulses in parallel. This process of applying SET pulses was repeated at every time instance. The maximum SET current applied to the devices during the experiment was 80 μA.

As described earlier, owing to the temporal correlation between the processes, the devices assigned to those processes are expected to go to a high conductance state. We periodically read the conductance values of all PCM devices using the on-chip read circuitry and the on-chip analog-to-digital convertor (ADC). The resulting map of the conductance values is shown in Fig. 5b. Also shown is the corresponding distribution of the conductance values (Fig. 5c). This distribution shows that we can distinguish between the correlated and the uncorrelated processes. We constructed a binary classifier by slicing the histogram of Fig. 5c according to some threshold, above which processes are labeled correlated and below which processes are labeled uncorrelated. The threshold parameter can be swept across the domain, resulting in an ensemble of different classifiers, each with its own statistical characteristics (e.g., precision and recall). The area under the precision-recall curve (AUC) is an excellent metric for quantifying the performance of the classifier. The AUC is 0.93 for the computational memory approach compared to 0.095 for a random classifier that simply labels processes as correlated with some arbitrary probability. However, the performance is still short of that of an ideal classifier with AUC equal to one and this is attributed to the variability and conductance fluctuations discussed earlier. However, it is remarkable that in spite of these issues, we are able to perform the correlation detection with significantly high accuracy. Note that there are several applications, such as sensory data processing, where these levels of accuracy would be sufficient. Moreover, we could improve the accuracy by using multiple devices to interface with a single random process and by averaging their conductance values. This concept is also illustrated in the experimental demonstration on weather data that is described next. The conductance fluctuations can also be minimized using concepts such as projected phase change memory^34^.

Note that the correlations need to be detected within a certain period of time. This arises from the finite conductance range of the PCM devices. There is a limit to the *u*_a_ and hence the maximum conductance values that the devices can achieve. The accumulation curves in Fig. 4d clearly show that the mean conductance values begin to saturate after the application of a certain number of pulses. If the correlations are not detected within a certain amount of time, the conductance values corresponding to the correlated processes saturate while those corresponding to the uncorrelated processes continue to increase. Once the correlations have been detected, the devices need to be RESET, and the operation has to be resumed to detect subsequent correlations. The application of shorter SET pulses is one way to increase this time period. The use of multiple devices to interface with the random processes can also increase the overall conductance range.

As per Eq. 6, we would expect the level of separation between the distributions of correlated and uncorrelated groups to increase with increasing values of the correlation coefficient. We could confirm experimentally that the correlated groups can be detected down to very low correlation coefficients such as *c *= 0.01 (Supplementary Note 2, Supplementary Movie 1 and Supplementary Movie 2). We also quantified the performance of the binary classifier by obtaining the precision-recall curves and could show that in all cases, the classifiers performed significantly better than a baseline, random classifier (Supplementary Fig. 2). Experiments also show that there is a potential for this technique to be extended to detect multiple correlated groups having different correlation coefficients (Supplementary Note 3).

### Experimental demonstration with weather data

A second demonstration is based on real-world data from 270 weather stations across the USA. Over a period of 6 months, the rainfall data from each station constituted a binary stochastic process that was applied to the computational memory at one-hour time steps. The process took the value 1 if rainfall occurred in the preceding one-hour time window, else it was 0 (Fig. 5d). An analysis of the uncentered covariance matrix shows that several correlated groups exist and that one of them is predominant. As expected, also a strong geographical correlation with the rainfall data exists (Fig. 5e). Correlations between the rainfall events are also reflected in the geographical proximity between the corresponding weather stations. To detect the predominant correlated group using computational memory, we used the same approach as above, but with 4 PCM devices interfacing with each weather station data. The four devices were used to improve the accuracy. At each instance in time, the SET current was calculated to be equal to $0.0013 \times M(k)$ μA. Next, the PCM chip was instructed to program the 270 × 4 devices sequentially with the calculated SET current. The on-chip write circuitry applies a write pulse with the calculated SET current to all PCM devices for which **X**_*i*_(*k*) = 1. We chose not to program the devices if the SET current was less than 25 μA. The duration of the pulse was fixed to be 50 ns, and the maximum SET current applied to the devices was 80 μA. The resulting device conductance map (averaged over the four devices per weather station) shows that the conductance values corresponding to the predominant correlated group of weather stations are comparably higher (Fig. 5f).

Based on a threshold conductance value chosen to be 2 μS, we can classify the weather stations into correlated and uncorrelated weather stations. This conductance threshold was chosen to get the best classifier performance (see Supplementary Note 2). We can also make comparisons with established unsupervised classification techniques such as *k*-means clustering. It was seen that, out of the 270 weather stations, there was a match for 245 weather stations. The computational memory approach classified 12 stations as uncorrelated that had been marked correlated by the *k*-means clustering approach. Similarly, the computational memory approach classified 13 stations as correlated that had been marked uncorrelated by the *k*-means clustering approach. Given the simplicity of the computational memory approach, it is remarkable that it can achieve this level of similarity with such a sophisticated and well-established classification algorithm (see Supplementary Note 4 for more details).

## Discussion

The scientific relevance of the presented work is that we have convincingly demonstrated the ability of computational memory to perform certain high-level computational tasks in a non-von Neumann manner by exploiting the dynamics of resistive memory devices. We have also demonstrated the concept experimentally at the scale of a million PCM devices. Even though we programmed the devices sequentially in the experimental demonstrations using the prototype chip, we could also program them in parallel provided there is a sufficient number of write modules. A hypothetical computational memory module performing correlation detection need not be substantially different from conventional memory modules (Supplementary Note 5). The main constituents of such a module will also be a memory controller and a memory chip. Tasks such as computing *M*(*k*) can easily be performed in the memory controller. The memory controller can then convey the write/read instructions to the memory chip.

In order to gain insight into the potential advantages of a correlation detector based on computational memory, we have compared the hypothetical performance of such a module with that of various implementations using state-of-the-art computing hardware (Supplementary Note 6). For this study, we have designed a multi-threaded implementation of correlation detection, an implementation that can leverage the massive parallelism offered by graphical processing units (GPUs), as well as a scale-out implementation that can run across several GPUs. All implementations were compiled and executed on an IBM Power System S822LC system. This system has two POWER8 CPUs (each comprising 10 cores) and 4 Nvidia Tesla P100 graphical processing units (attached using the NVLink interface). A detailed profiling of the GPU implementation reveals two key insights. Firstly, we find that the fraction of time computing the momentum *M*(*k*) is around $2\%$ of the total execution time. Secondly, we observe that the performance is ultimately limited by the memory bandwidth of the GPU device. We then proceed to estimate the time that would be needed to perform the same task using a computational memory module: we determine the time required to compute the momentum on the memory controller, as well as the additional time required to perform the in-memory part of the computation. We conclude that by using such a computational memory module, one could accelerate the task of correlation detection by a factor of 200 relative to an implementation that uses 4 state-of-the-art GPU devices. We have also performed power profiling of the GPU implementation, and conclude that the computational memory module would provide a significant improvement in energy consumption of two orders of magnitude (Supplementary Note 6).

An alternative approach to using PCM devices will be to design an application-specific chip where the accumulative behavior of PCM is emulated using complementary metal-oxide semiconductor (CMOS) technology using adders and registers (Supplementary Note 7). However, even at a relatively large 90 nm technology node, the areal footprint of the computational phase change memory is much smaller than that of CMOS-only approaches, even though the dynamic power consumption is comparable. By scaling the devices to smaller dimensions and by using shorter write pulses, these gains are expected to increase several fold^35,36^. The ultra-fast crystallization dynamics and non-volatility ensure a multi-time scale operating window ranging from a few tens of nanoseconds to years. These attributes are particularly attractive for slow processes, where the leakage of CMOS would dominate the dynamic power because of the low utilization rate.

It can be shown that a single-layer spiking neural network can also be used to detect temporal correlations^30^. The event-based data streams can be translated into pre-synaptic spikes to a synaptic layer. On the basis of the synaptic weights, the postsynaptic potentials are generated and added to the membrane potential of a leaky integrate and fire neuron. The temporal correlations between the pre-synaptic input spikes and the neuronal-firing events result in an evolution of the synaptic weights due to a feedback-driven competition among the synapses. In the steady state, the correlations between the individual input streams can be inferred from the distribution of the synaptic weights or the resulting firing activity of the postsynaptic neuron. Recently, it was shown that in such a neural network, PCM devices can serve as the synaptic elements^37,38^. One could argue that the synaptic elements serve as some form of computational memory. Even though both approaches aim to solve the same problem, there are some notable differences. In the neural network approach, it is the spike-timing-dependent plasticity rule and the network dynamics that enable correlation detection. One could use any passive multi-level storage element to store the synaptic weight. Also note that the neuronal input is derived based on the value of the synaptic weights. It is challenging to implement such a feedback architecture in a computational memory unit. Such feedback architectures are also likely to be much more sensitive to device variabilities and nonlinearities and are not well suited for detecting very low correlations^37,39^.

Detection of statistical correlations is just one of the computational primitives that could be realized using the crystallization dynamics. Another application of crystallization dynamics is that of finding factors of numbers, which we referred to in the introduction^20^. Assume that a PCM device is initialized in such a way that after the application of *X* number of pulses, the conductance exceeds a certain threshold. To check whether *X* is a factor of *Y*, *Y* number of pulses are applied to the device, re-initializing the device each time the conductance exceeds the threshold. It can be seen that if after the application of *Y* pulses, the conductance of the device is above the threshold, then *X* is a factor of *Y*. Another fascinating application of crystallization dynamics is to realize matrix–vector multiplications. To multiple an *N *× *N* matrix, *A*, with a *N* × 1vector, *x*, the elements of the matrix and the vector can be translated into the durations and amplitudes of a sequence of crystallizing pulses applied to an array of *N* PCM devices. It can be shown that by monitoring the conductance levels of the PCM devices, one obtains a good estimate of the matrix–vector product (Supplementary Note 8). Note that such an approach consumes only *N* devices compared to the existing approach based on the Kirchhoff’s circuit laws that requires *N* × *N* devices.

In addition to the crystallization dynamics, one could also exploit other rich dynamic behavior in PCM devices, such as the dynamics of structural relaxation. Whenever an amorphous state is formed via the melt-quench process, the resulting unstable glass state relaxes to an energetically more favorable ideal glass state^25,40–42^ (Supplementary Note 9). This structural relaxation, which codes the temporal information of the application of write pulses, can be exploited to perform tasks such as the detection of rates of processes in addition to their temporal correlations (Supplementary Note 9). It is also foreseeable that by further coupling the dynamics of these devices, we can potentially solve even more intriguing problems. Suggestions of such memcomputing machines that could solve certain non-deterministic polynomial (NP) problems in polynomial (P) time by exploiting attributes, such as the inherent parallelism, functional polymorphism, and information overhead are being actively investigated^43,44^. The concepts presented in this work could also be extended to the optical domain using devices such as photonic PCM^45^. In such an approach, optical signals instead of electrical signals will be used to program the devices. These concepts are also not limited to PCM devices: several other memristive device technologies exist that possess sufficiently rich dynamics to serve as computational memory^46^. However, it is worth noting that PCM technology is arguably the most advanced resistive memory technology at present with a very well-established multi-level storage capability^21^. The read endurance is assumed to be unlimited. There are also recent reports of more than 10^12^ RESET/SET endurance cycles^47^. Note that in our experiments, we mostly apply only the SET pulses, and in this case the endurance is expected to be substantially higher.

To summarize, the objective of our work was to realize a high-level computational primitive or machine-learning algorithm using computational memory. We proposed an algorithm to detect temporal correlations between event-based data streams that exploits the crystallization dynamics of PCM devices. The conductance of the PCM devices receiving correlated inputs evolves to a high value, and by monitoring these conductance values we can detect the temporal correlations. We performed a large-scale experimental demonstration of this concept using a million PCM devices, and could successfully detect weakly correlated processes in artificially generated stochastic input data. This experiment demonstrates the efficacy of this concept even in the presence of device variability and other non-ideal behavior. We also successfully processed real-world data sets from weather stations in the United States and obtained classification results similar to the *k*-means clustering algorithm. A detailed comparative study with respect to state-of-the-art von Neumann computing systems showed that computational memory could lead to orders of magnitude improvements in time/energy-to-solution compared to conventional computing systems.

## Methods

### Phase change memory chip

The PCM devices were integrated into the chip in 90 nm CMOS technology^32^. The phase change material is doped Ge_2_Sb_2_Te_2_ (d-GST). The bottom electrode has a radius of approx. 20 nm and a length of approx. 65 nm, and was defined using a sub-lithographic key-hole transfer process^48^. The phase change material is approx. 100 nm thick and extends to the top electrode. Two types of devices are available on-chip. They differ by the size of their access transistor. The first sub-array contains 2 million devices. In the second sub-array, which contains 1 million devices, the access transistors are twice as large. All experiments in this work were done on the second sub-array, which is organized as a matrix of 512 word lines (WL) and 2048 bit lines (BL). The selection of one PCM device is done by serially addressing a WL and a BL. A single selected device can be programmed by forcing a current through the BL with a voltage-controlled current source. For reading a PCM cell, the selected BL is biased to a constant voltage of 200 mV. The resulting read current is integrated by a capacitor, and the resulting voltage is then digitized by the on-chip 8-bit cyclic ADC. The total time of one read is 1 *μ*s. The readout characteristic is calibrated by means of on-chip reference poly-silicon resistors.

### Generation of 1M random processes and experimental details

Let **X**_*r*_ be a discrete binary process with probabilities *P*(*X*_*r*_(*k*) = 1) = *p* and *P*(*X*_*r*_(*k*) = 0) = 1 − *p*. Using **X**_*r*_ as the reference process, *N* binary processes can be generated via the stochastic functions^39^

10 $$\theta = P\left( {X_i(k) = 1\left| {X_r(k)} \right. = 1} \right) = p + \sqrt c \left( {1 - p} \right)$$

11 $$\phi = P\left( {X_i(k) = 1\left| {X_r(k)} \right. = 0} \right) = p\left( {1 - \sqrt c } \right)$$

12 $$P\left( {X_i(k) = 0} \right) = 1 - P\left( {X_i(k) = 1} \right).$$

It can be shown that *E*(*X*_*i*_(*k*)) = *p* and *Var*(*X*_*i*_(*k*)) = *p*(1 − *p*). If two processes **X**_*i*_ and **X**_*j*_ are both generated using Eqs. 10–12, then the expectation of their product is given by:

$$\begin{array}{ccccc}\\ E\left( {X_i\left( k \right)X_j\left( k \right)} \right) = P\left( {X_i\left( k \right) = 1,X_j\left( k \right) = 1} \right)\\ \\ = \mathop {\sum}\limits_{v \in \{ 0,1\} } {P\left( {X_i\left( k \right) = 1,X_j\left( k \right) = 1\left| {X_r\left( k \right)} \right. = v} \right)} P\left( {X_r\left( k \right) = v} \right).\\ \end{array}$$

Conditional on the value of the process **X**_*r*_, the two processes **X**_*i*_ and **X**_*j*_ are statistically independent by construction, and thus the conditional joint probability *P*(*X*_*i*_(*k*) = 1, *X*_*j*_(*k*) = 1|*X*_*r*_(*k*) = *v*) can be factorized as follows:

$$\begin{array}{ccccc}\\ E\left( {X_i(k)X_j(k)} \right) = \mathop {\sum}\limits_{v \in \{ 0,1\} } P\left( {X_i(k) = 1\left| {X_r(k)} \right. = v} \right)P\left( {X_j(k) = 1\left| {X_r(k)} \right. = v} \right)\\ P\left( {X_r(k) = v} \right)\\ \\ = \theta ^2p + \phi ^2(1 - p)\\ \\ = p^2 + cp(1 - p),\\ \end{array}$$

where the final equality is obtained by substituting the preceding expressions for *θ* and *ϕ*, followed by some simple algebraic manipulation. It is then straightforward to show that the correlation coefficient between the two processes is equal to *c* as shown below:

13 $$\begin{array}{ccccc}\\ Cov\left( {X_i(k)X_j(k)} \right) = E\left( {X_i(k)X_j(k)} \right) - E\left( {X_i(k)} \right)E\left( {X_j(k)} \right)\\ \\ = p^2 + cp(1 - p) - p^2\\ \\ \frac{{Cov(X_i(k)X_j(k))}}{{\sqrt {Var(X_i)Var(X_j)} }} = c\\ \end{array}$$

For the experiment presented, we chose an **X**_*r*_ where *p* = 0.01. A million binary processes were generated. Of these, *N*_*c*_ = 95,525 are correlated with *c* > 0. The remaining 904,475 processes are mutually uncorrelated. Each process is mapped to one pixel of a 1000 × 1000 pixel black-and-white sketch of Alan Turing: white pixels are mapped to the uncorrelated processes; black pixels are mapped to the correlated processes. The seemingly arbitrary choice of *N*_*c*_ arises from the need to match with the black pixels of the image. The pixels turn on and off in accordance with the binary values of the processes. One phase change memory device is allocated to each of the one million processes.

### Weather data-based processes and experimental details

The weather data was obtained from the National Oceanic and Atmospheric Administration (http://www.noaa.gov/) database of quality-controlled local climatological data. It provides hourly summaries of climatological data from approximately 1600 weather stations in the United States of America. The measurements were obtained over a 6-month period from January 2015 to June 2015 (181 days, 4344 h). We generated one binary stochastic process per weather station. If it rained in any given period of 1 h in a particular geographical location corresponding to a weather station, then the process takes the value 1; else it will be 0. For the experiments on correlation detection, we picked 270 weather stations with similar rates of rainfall activity.

### Data availability

The data that support the findings of this study are available from the corresponding author upon request.

## Electronic supplementary material

Supplementary Information

Description of Additional Supplementary Files

Supplementary Movie 1

Supplementary Movie 2
