# Supplementary material for: Temporal correlation detection using computational phase-change memory
Source: Nat Commun. 2017 Oct 24;8:1115. doi: 10.1038/s41467-017-01481-9 (PMC5653661; doi:10.1038/s41467-017-01481-9)
Supplement: Supplementary file 1 — Supplementary Information [file 41467_2017_1481_MOESM1_ESM.pdf]

SUPPLEMENTARY NOTE 1: METHODOLOGY FOR DETECTING TEMPORAL CORRELATIONS

This note provides additional details on the technique to detect correlations between random processes. Let $\mathbf{X}_i = \{X_i(k)\}$ be a discrete-time binary stochastic process. Then $X_i(k)$ is a random variable with probabilities

$$P[X_i(k) = 1] = p \tag{1}$$

$$P[X_i(k) = 0] = 1 - p, \tag{2}$$

for $0 \le p \le 0.5$. It can be shown that

$$E[X_i(k)] = p \tag{3}$$

$$\text{Var}[X_i(k)] = p(1-p). \tag{4}$$

Let $\mathbf{X}_i$ and $\mathbf{X}_j$ be discrete-time binary stochastic processes with the same value of parameter p. Then the correlation coefficient of the random variables $X_i(k)$ and $X_j(k)$ at time instant k is defined as

$$\begin{aligned} c &= \frac{\text{Cov}[X_i(k), X_j(k)]}{\sqrt{\text{Var}[X_i(k)]\text{Var}[X_j(k)]}} \\ &= \frac{E[X_i(k)X_j(k)] - p^2}{p(1-p)}. \end{aligned} \tag{5}$$

From Equation (5), $E[X_i(k)X_j(k)]$ denoted by $R_{ij}(k)$ is given by

$$R_{ij}(k) = p^2 + cp(1-p). \tag{6}$$

Let $k \in \{1, 2, 3, \dots K\}$, then for stationary ergodic processes, an estimate of R_{ij} which is independent of k is given by

$$\hat{R}_{ij} = \frac{1}{K}\sum_{k=1}^{K} X_i(k)X_j(k). \tag{7}$$

It can easily be seen that

$$\begin{aligned} R_{ij} &= E[\hat{R}_{ij}] \\ &= \frac{1}{K}\sum_{k=1}^{K} E[X_i(k)X_j(k)] \\ &= \begin{cases} p^2 + cp(1-p), & \text{for } i \neq j \\ p, & \text{for } i = j. \end{cases} \end{aligned} \tag{8}$$

Assume that there are N such discrete-time binary processes, of which N_c are correlated. Moreover, let us define $\hat{W}_i = \sum_{j=1}^{N} \hat{R}_{ij}$. From Equation (8), it can be shown that if $\mathbf{X}_i$ belongs to the correlated group with correlation coefficient $c > 0$, then

$$E[\hat{W}_i] = (N-1)p^2 + p + (N_\text{c} - 1)cp(1-p). \tag{9}$$

In contrast, if $\mathbf{X}_i$ belongs to the uncorrelated group, then

$$E[\hat{W}_i] = (N-1)p^2 + p. \tag{10}$$

It is possible to show that the variance of the estimator $\hat{W}_i$ decreases as the number of time steps K grows:

$$\begin{aligned} \text{Var}[\hat{W}_i] &= E[\hat{W}_i^2] - E[\hat{W}_i]^2 \\ &= \frac{1}{K}\sum_{j=1}^{N}\sum_{j'=1}^{N} \text{Cov}[X_i(k)X_j(k), X_i(k)X_{j'}(k)] \\ &\le \frac{1}{K}\sum_{j=1}^{N}\sum_{j'=1}^{N} \sqrt{\text{Var}[X_i(k)X_j(k)]\text{Var}[X_i(k)X_{j'}(k)]} \le \frac{N^2}{4K}. \end{aligned} \tag{11}$$

Hence by monitoring $\hat{W}_i$ in the limit of large K, we can determine which processes are correlated with $c > 0$. Moreover, it can be seen that with increasing c and N_c, it becomes easier to determine whether a process belongs to a correlated group.

SUPPLEMENTARY NOTE 2: DEPENDENCE ON THE CORRELATION COEFFICIENT

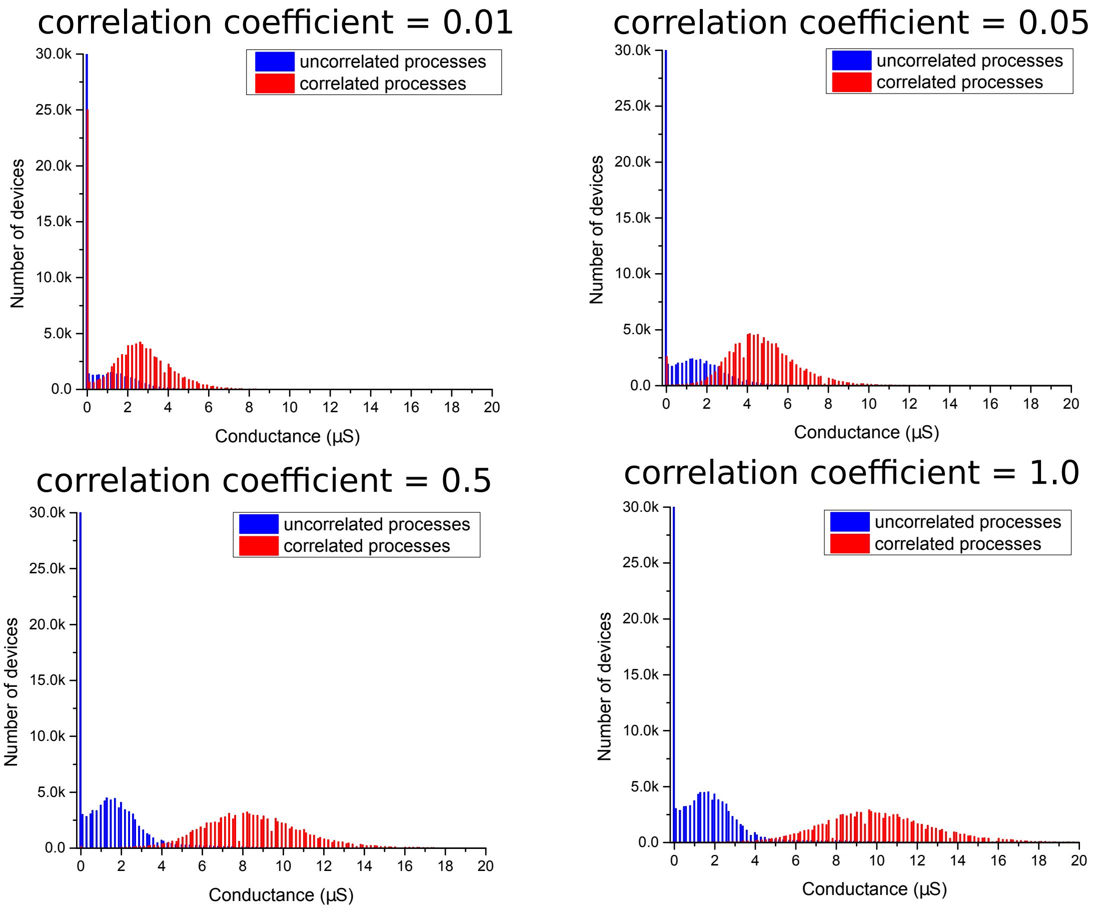

Supplementary Figure 1. **Dependence on the correlation coefficient.** Histograms showing the distribution of device conductance values at the end of the experiment for different values of c. The separation between the correlated and the uncorrelated groups increases with increasing values of c.

As discussed in Supplementary Note 1, the ability to detect temporal correlations heavily depends on the extent of correlation. For example, one would expect that the task becomes progressively easier with increasing correlation coefficient. Experimental results indeed show this trend. Experiments were performed with 1 million processes in total. Of these, we arbitrarily chose a subset of 95,525 processes to be mutually correlated with an instantaneous correlation coefficient, c. The remaining 904,475 processes are uncorrelated. Supplementary Figure 1 shows distribution of the device conductance values at the end of the experiment for different values of c. It can be seen that with increasing values of c, there is a larger separation between the correlated and uncorrelated groups.

To perform correlation detection, we construct a binary classifier by slicing the histograms of Supplementary Figure 1 according to some threshold, above which processes are labelled correlated and below which processes are labelled uncorrelated. The threshold parameter can be swept across the domain, resulting in an ensemble of different classifiers, each with its own statistical characteristics (e.g., precision and recall). In Supplementary Figure 2, we plot the precision–recall curves for such an ensemble for increasing values of the correlation coefficient, c. We have opted to study the precision–recall curves rather than the corresponding receiver operator characteristic (ROC) curves because it is well established that precision–recall analysis is better suited for measuring the classification performance on imbalanced datasets[1]. As a baseline, we also present the precision–recall curve for the random classifier that simply labels processes as correlated with some arbitrary probability. As expected, we observe an increasingly better quality of classification (i.e., more area under the precision–recall curve) as the correlation coefficient increases. In all cases, the area under the curve is significantly larger than that of the baseline, random classifier.

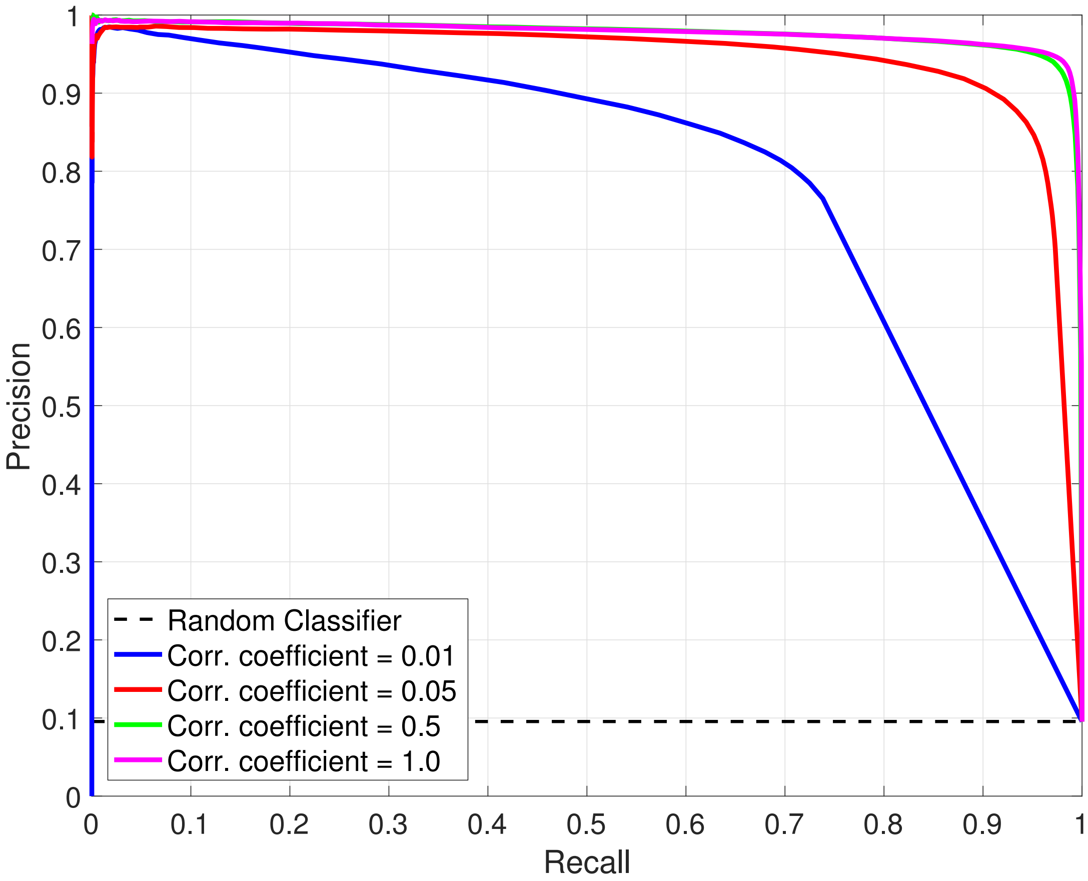

Supplementary Figure 2. Precision–recall curves as a function of correlation coefficient.

SUPPLEMENTARY NOTE 3: DETECTING MULTIPLE CORRELATIONS

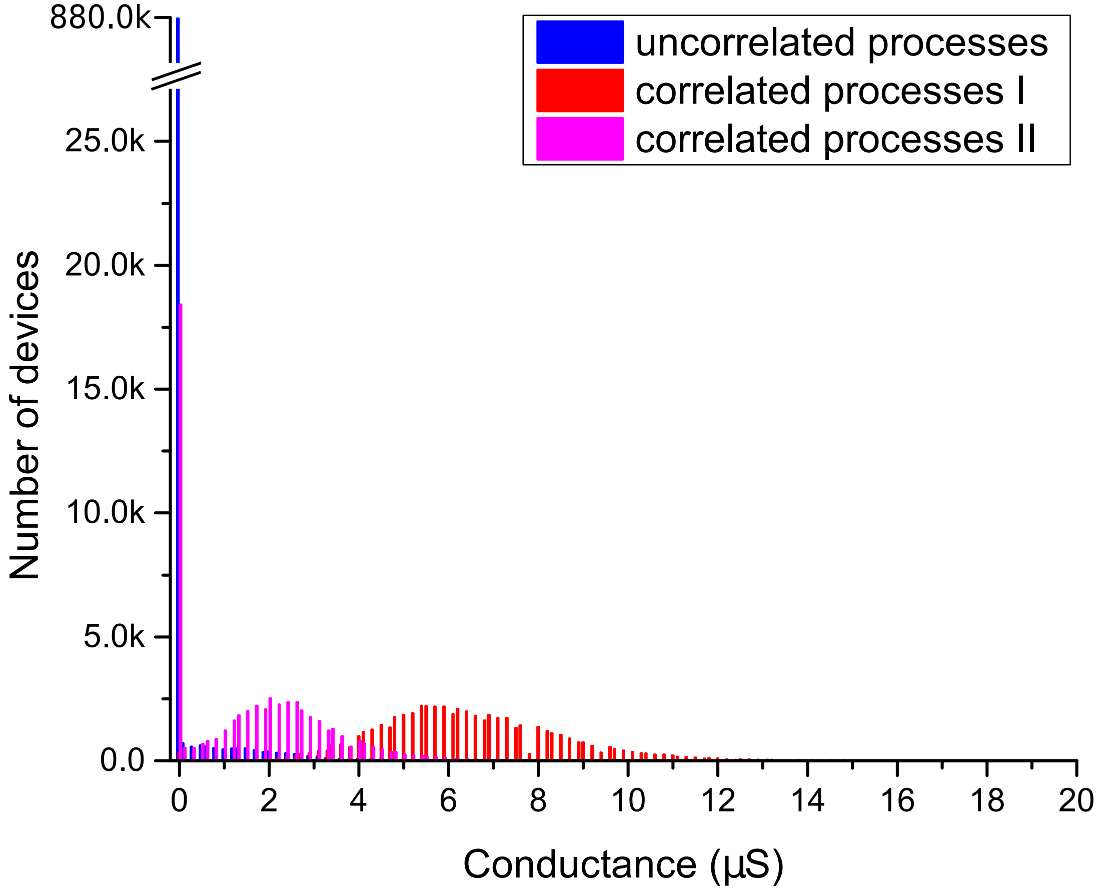

Supplementary Figure 3. **Detecting multiple correlations.** The distribution of conductance levels after 2455 time steps clearly shows that the algorithm is able to detect multiple correlations.

It is also possible to detect multiple correlations using the computational memory approach. To show this, an experiment is presented with 1 million processes in total. Of these 889,007 are uncorrelated. 56,296 processes are correlated with a correlation coefficient of 0.05, whereas 54,697 processes are correlated with a correlation coefficient of 0.08. The sets of correlated processes are mutually uncorrelated. Each of the processes is assigned to a single PCM device. The resulting distribution of conductance levels after 2455 time steps is shown in Supplementary Figure 3. One can clearly see the difference in the conductance levels associated with the devices assigned to the two different correlated groups.

SUPPLEMENTARY NOTE 4: COMPARISON WITH k-MEANS CLUSTERING ALGORITHM

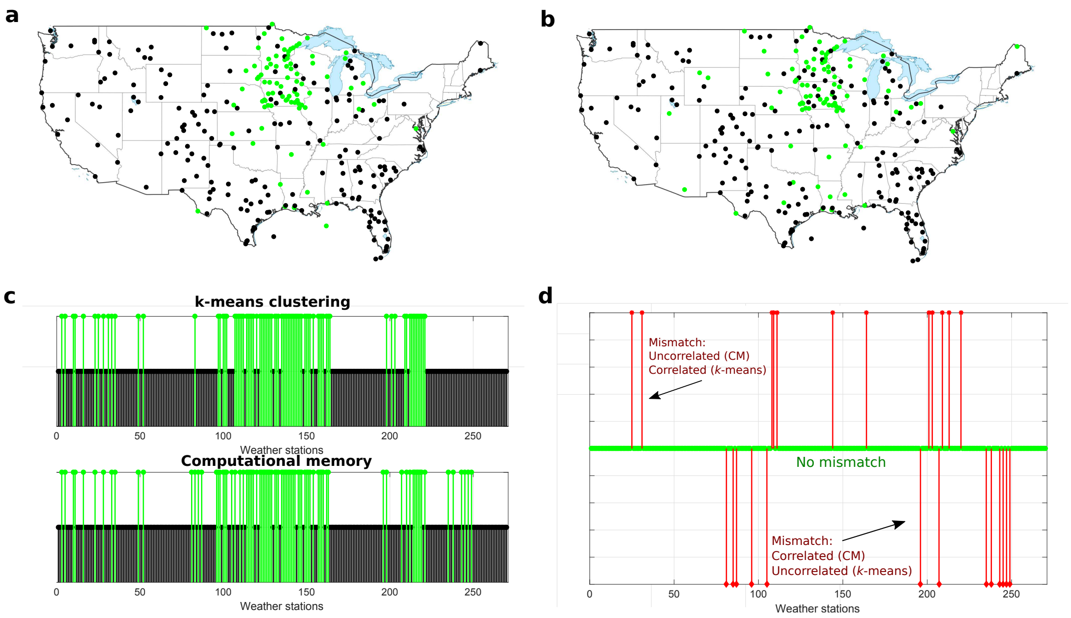

Supplementary Figure 4. **Comparison with k-means clustering algorithm.** (**a**) The k-means clustering algorithm was used to cluster the weather stations into a correlated and an uncorrelated group. In the resulting map of the United States, the correlated weather stations are denoted in green and the uncorrelated ones in black. (**b**) The computational memory approach was also used to group the weather stations into a correlated and an uncorrelated group. In the resulting map of the United States, the correlated weather stations are denoted in green and the uncorrelated ones in black. (**c**) Out of the 270 weather stations, there was agreement between the two approaches for 245 weather stations. (**d**) The computational memory approach classified 12 weather stations as uncorrelated that had been marked correlated by the k-means clustering approach. Similarly, the computational memory approach classified 13 weather stations as correlated that had been marked uncorrelated by the k-means clustering approach.

For the experiment using the weather data, another way to classify the processes based on their temporal correlation is via the k-means clustering algorithm. Consider a set of observations $(x_1, x_2, \ldots, x_n)$, where each observation $x_i \in \mathbb{R}^d$, and a k-fold partitioning of the same observations $\mathcal{S} = (S_1, S_2, \ldots, S_n)$. Each individual partition S_i is a set referred to as a cluster. The optimal solution to the k-means clustering problem is given by the partitioning $\hat{\mathcal{S}}$ that satisfies the following:

$$\hat{S} = \underset{S}{\operatorname{argmin}} \sum_{i=1}^{k} \sum_{x \in S_i} ||x - \mu_i||^2, \tag{12}$$

where $\mu_i \in \mathbb{R}^d$ denotes the cluster mean (or centroid). While the k-means problem has been proved to be NP-hard[2], heuristic techniques such as Lloyd's algorithm[3] are often used to find approximate solutions. Lloyd's algorithm begins by initializing each cluster centroid by selecting one of the n observations uniformly at random. It then proceeds to pass through the n observations, assigning each observation to the cluster that is closest in the sense of Euclidean distance from the observation vector to the cluster centroid. At this end of this pass, each cluster centroid is updated by computing the mean of all of the observations that were assigned to it in the preceding pass. This process is repeated for a fixed number of iterations, T, or until some convergence criterion is met. As the algorithm can converge to a local optimum, it is common practice to perform a number of outer iterations using different initial centroids to see whether a better solution can be obtained. The complexity of a single outer iteration of Lloyd's algorithm has complexity $\mathcal{O}(ndkT)$. For structured data, one typically finds that the algorithm converges with relatively few inner iterations. However, it has been shown that the algorithm can need $T = 2^{\Omega(\sqrt{n})}$ iterations in the worst case and thus exhibits super-polynomial complexity[4].

Here, we compare the k-means clustering approach with the computational memory approach. The k-means clustering algorithm was used to cluster the weather stations into two groups based on their Euclidean distance in the $\mathbb{R}^K$ space. The cluster of the weather stations with lesser mean distance from each other was denoted the correlated group, and the other cluster was denoted the uncorrelated group. Supplementary Figure 4(a) shows the map of the United States with the correlated weather stations denoted in green and the uncorrelated ones in black.

The computational memory approach was also used to group the weather stations into a correlated and an uncorrelated group. As described in the main text, we used 4 devices to interface with a single weather station. If the mean conductance value

exceeds 2 μS, then that weather station was considered to belong to the correlated group. The resulting map of the United States with the correlated weather stations as detected by the computational memory approach is shown in Supplementary Figure 4(b).

Out of the 270 weather stations, there was agreement between the two approaches for 245 weather stations (Supplementary Figure 4(c)). The computational memory approach classified 12 weather stations as uncorrelated that had been marked correlated by the k-means clustering approach. Similarly, the computational memory approach classified 13 weather stations as correlated that had been marked uncorrelated by the k-means clustering approach (Supplementary Figure 4(d)). Given the simplicity of the computational memory approach, it is remarkable that it can achieve this level of similarity with such a sophisticated classification algorithm. Also note that, given the difference between the two algorithms (covariance matrix-based and k-means clustering), calculations show that the expectation was to get an agreement only for 251 weather stations. These experiments also revealed the advantages of having multiple devices interfacing to a single random process. For example, if we had used only one device per weather station, our accuracies would have been lower and the agreement with the k-means approach would have been obtained only for 229 weather stations.

SUPPLEMENTARY NOTE 5: A HYPOTHETICAL COMPUTATIONAL PHASE-CHANGE MEMORY UNIT

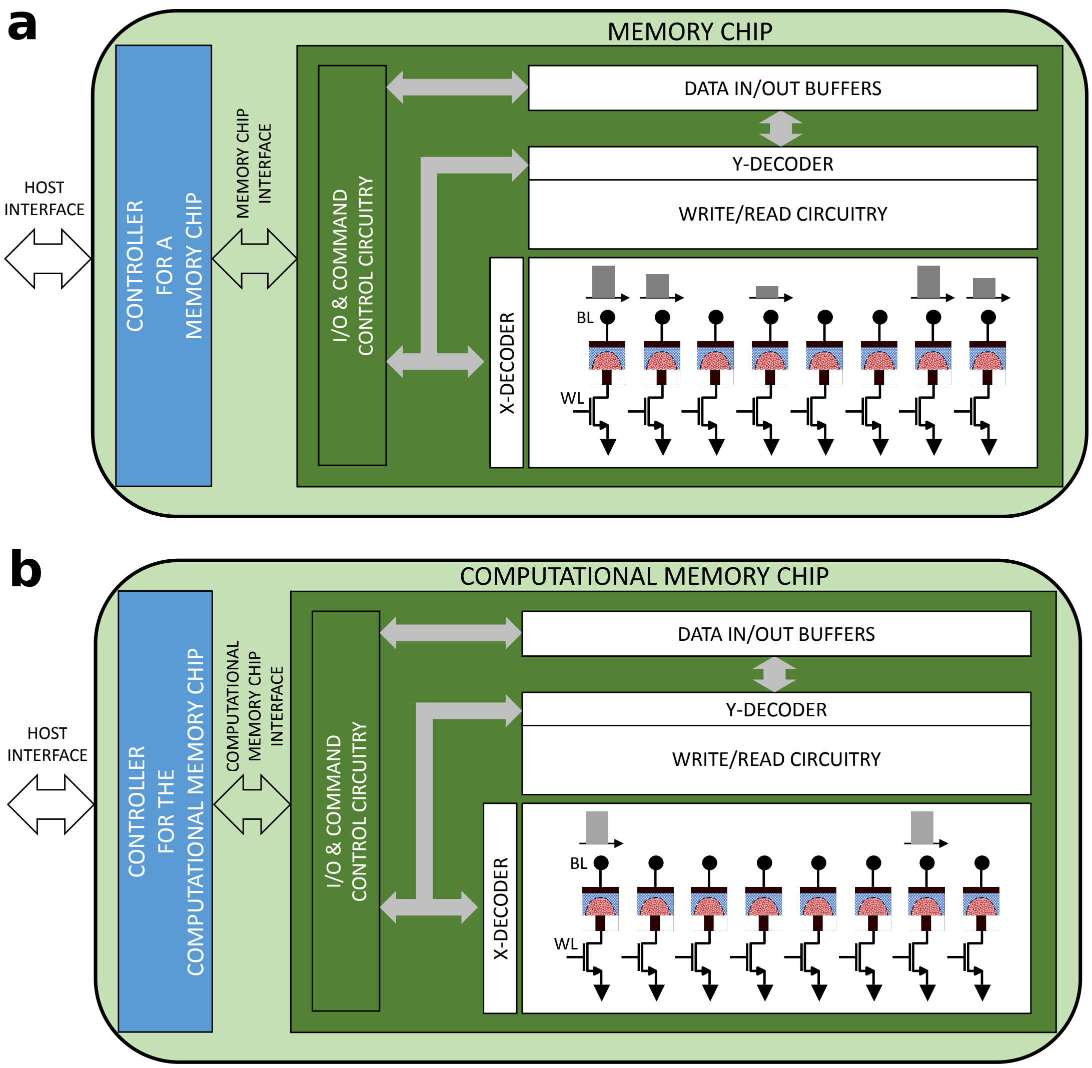

Supplementary Figure 5. **Schematic representation of the various building blocks.** (**a**) Conventional PCM-based memory unit and (**b**) hypothetical computational phase-change memory unit. BL stands for Bit Line and WL for Word Line.

Here we present some details as to how a hypothetical computational phase-change memory unit would look. In particular, we would like to highlight the similarity of such a computational memory chip with a conventional PCM-based memory unit. Supplementary Figure 5(a) presents a schematic representation of the various building blocks of the conventional memory unit. The main constituents are a memory controller chip and a memory chip. The memory unit typically interfaces to a host via a host interface. During the write operation, the controller may apply some type of encoding to the data before sending the corresponding write commands to the memory based on the Input/Output (I/O) interface of the chip. During the read operation, the data received from the memory chip is decoded and error correction may be performed before the data is passed to the host. The memory chip comprises the I/O and command control circuitry, the I/O data buffers, the address-decoding circuitry, the read/write circuitry and the memory array. During the write operation, the data is loaded to the input buffers and the write circuity will be invoked to program the memory devices in parallel or sequentially. The devices can be addressed directly or in an incremental fashion (Bit Lines and World Lines can be used to access an individual memory cell or a group of memory cells). During the read operation, the memory cells are read in parallel or sequentially, and the digitized information is passed on to the memory controller.

Supplementary Figure 5(b) presents a schematic representation of the various building blocks of a potential computational memory unit. Here again, the main constituents are a memory controller chip and a memory chip, and the chip can interface to a host via an appropriate host interface. For example, the event-based data streams arrive at the computational memory chip via this interface. Based on this, the instantaneous collective momentum can be calculated in the controller. A digital bit sequence

that indicates the programming current or pulse width corresponding to the collective momentum is passed to the memory chip along with the addressing information of the memory devices that need to be programmed. The bit sequence and addressing information are formatted according to some encoding scheme determined by the memory chip interface. The devices that need to be programmed will be programmed with the same programming conditions, whereas the devices that need not be programmed can be indicated using a designated bit sequence.

SUPPLEMENTARY NOTE 6: COMPARISON WITH IMPLEMENTATIONS WITH CPUS AND GPUS

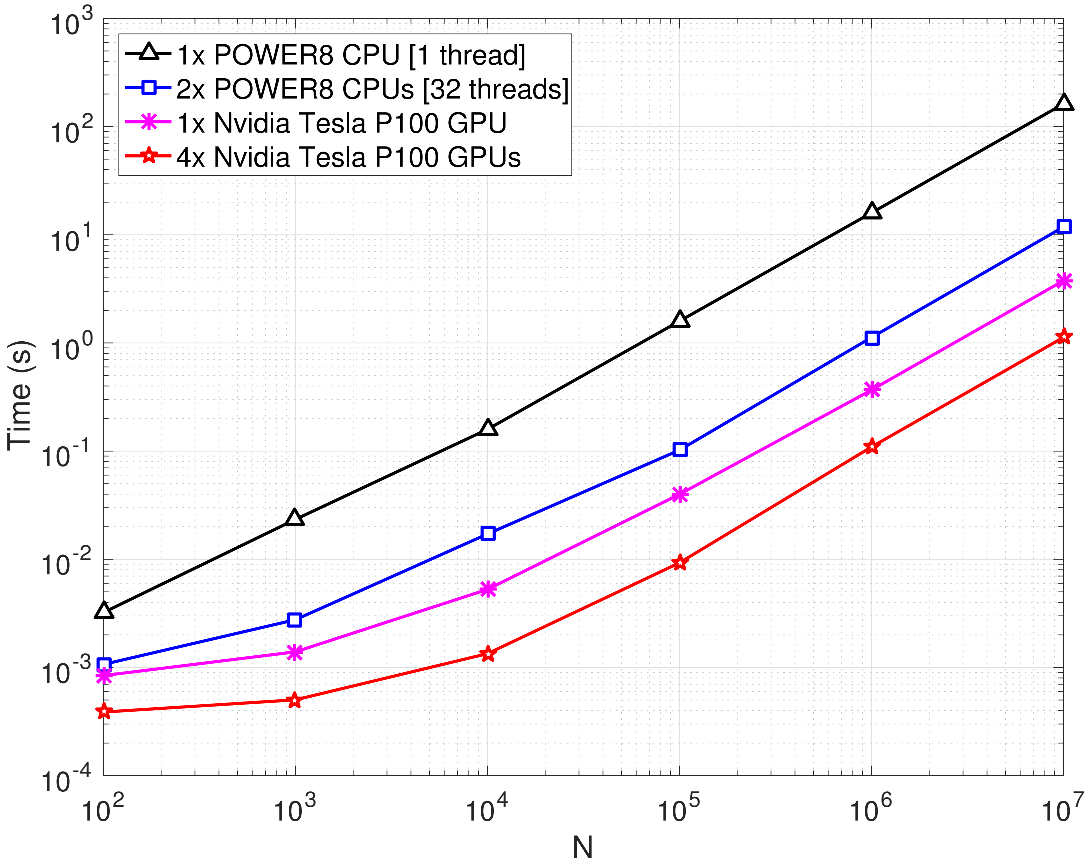

Supplementary Figure 6. Time required to compute the coefficients W_i for $K = 10^4$ for increasing number of processes N.

In this note we consider how one can efficiently compute the coefficients W_i using state-of-the-art CPU and GPU hardware and how such implementations compare with the proposed approach that uses computational memory. For a set for binary processes $X_i(k)$, the coefficient W_i can be computed as follows:

$$W_i = \sum_{k=1}^{K} X_i(k) \sum_{j=1}^{N} X_j(k) = \sum_{k=1}^{K} X_i(k) M(k) \quad (13)$$

The samples from all N processes at a given time step k are stored contiguously in memory, and a single bit is used to represent each sample $X_i(k)$, thus the samples from 32 different processes are packed inside one 4-byte unsigned integer data type. The total memory required to store the data at all K time steps is given by $KN/8$ bytes: for $K = 10^4$ and $N = 10^6$ this amounts to around 1.2 GB of data. The CPU-based implementation begins by initializing the estimates $W_i = 0$, for $i = 1, 2, \ldots, N$. The implementation then proceeds to iterate over the time steps $k = 1, 2, \ldots, K$. At each time step, we first compute the collective momentum $M(k)$: a 4-byte floating point number resulting from the summation of all bits $X_i(k)$ for $i = 1, 2, \ldots N$. To perform this addition operation, each bit $X_i(k)$ must first be extracted from the corresponding unsigned integer data type, and cast to a floating point number. Compiler flags were set to ensure that the additions are effectively vectorized, thus making full use of the arithmetic resources provided by the CPU. Once the collective momentum has been computed, we then perform a second pass through the processes, again casting each $X_i(k)$ to a floating point number, multiplying by $M(k)$, and adding the result to the current value of W_i. In this manner, the coefficients W_i are effectively accumulated in memory.

To harness the multi-core architecture of modern CPUs, the computation defined in equation (13) can easily be divided up amongst T threads. The time steps $k = 1, 2, \ldots, K$ are divided into T equal groups, and each thread is responsible for computing the part of the summation (13) corresponding to one of such groups. Once all threads have finished working, the partial results are reduced to obtain the final value for the coefficients W_i.

In a similar manner, the computation can be mapped to GPU hardware by launching a kernel function consisting of K thread blocks. Each thread block is responsible for the computation of a single summand in (13) and for the computation of

every summand in (13) (i.e., one thread block for every time step). The GPU schedules the thread blocks for execution on its streaming multi-processors as it sees fit. Each thread block computes the collective momentum $M(k)$ in a multi-threaded manner: each individual thread is responsible for only a part of the necessary summation over all processes, the partial results are stored in shared memory, and finally the results are reduced to obtain the resulting value for $M(k)$. The thread block then proceeds to pass over the processes again, this time multiplying each sample $X_i(k)$ by the momentum $M(k)$ and then using an atomic addition operation to accumulate the resulting values for W_i in the main memory of the GPU. It is possible to further distribute the computation across multiple GPUs by partitioning the time steps $k = 1, 2, \ldots, K$ into distinct groups, allowing each GPU to compute a partial result for the W_i for the set of time steps assigned to it, and then collecting and reducing the results from all GPUs at the end. As on the CPU, the binary data for the processes are packed into 4-byte unsigned integer data types, and all arithmetic is performed using 4-byte floating point data types. Thus, as before, each bit $X_i(k)$ must be unpacked and cast to a floating point number before it is used arithmetically.

To quantify the time required for such a calculation using state-of-the-art computing hardware, we measured the performance of various implementations that can be executed on an IBM* Power* System S822LC system. This system has 2 POWER8* CPUs (each comprising 10 cores) and 4 Nvidia Tesla P100 GPUs (attached using the NVLink interface). We measured the performance of a CPU-based implementation using only a single thread as well as of an implementation that uses 32 threads spread across the 2 CPUs. We also studied the performance of an implementation that uses a single GPU, as well as one that uses all 4 GPUs. The results are shown in Supplementary Figure 6 for $K = 10^4$ and increasing values of N. We observe linear scaling with N in all cases and a significant improvement from using the multi-threaded and GPU-based implementations. For instance, by using 4 GPUs, it was possible to compute all coefficients W_i for $N = 10^7$ processes in a single second.

To gain more insight into the computational complexity of the GPU-based correlation detector, in Supplementary Table 1 we present a profiling of the computational kernel that was obtained using the nvprof tool provided by NVIDIA. We present data for the experiment using a single P100 GPU and $N = 10^7$ processes with $K = 10^4$ time steps. We compare the resulting profiles for the case where the computational kernel only computes the collective momentum, with the numbers obtained for the full computational kernel as described above. Firstly, we note that while the kernel takes only 80 ms to compute the collective momentum, it requires 3.9 s to perform the full calculation (i.e., only 2% of the execution time is spent computing the collective momentum). Next, we note that when moving from the momentum-only kernel to the full kernel, most of the instruction counts either stay relatively constant or increase by a factor of two. There are three noticeable exceptions: the full kernel requires 102 billion floating point multiplications (the momentum computation requires zero), 26 billion global load transactions (only 800 million were necessary for the momentum computation) and 204 billion L2 transactions related to atomic requests (again, no atomic requests were necessary for the momentum computation). This huge increase in the number of L2 requests due to atomic operations leads to saturation of the GPU device's memory bandwidth and is thus the performance bottleneck for the kernel (this is confirmed by running the bottleneck analysis provided by NVIDIA's visual profiling tool).

To provide a point of comparison with the proposed approach that uses computational memory, we note that the time required to compute W_i for $N = 10^7$ processes will be dominated either by the time required to write to the PCM cells, t_1, or the time required to compute the collective momentum $M(k)$ on the memory controller, t_2. As all PCM cells can be programmed in parallel, the time spent writing to PCM is given by $t_1 = Kt_{\text{PCM}}$ where t_{PCM} is the PCM write latency. Assuming a write latency of 100 ns, we conclude that, for $K = 10^4$, the time spent writing to the PCM will be around 1 ms. Note that owing to the sparsity of the processes, only a fraction of the $N = 10^7$ devices receive a SET pulse, and hence the devices could be programmed in parallel. Even otherwise, it is possible to slightly offset the programming pulses in time without a significant penalty. The time t_2 is determined by how quickly one can count the number of ones in the binary sequence $(X_1(k), X_2(k), \ldots, X_N(k))$ on the memory controller. Assuming that one implements this addition using a tree structure in either an FPGA or an ASIC device, one can compute the collective momentum at a given time step in $L = \log_2(N)$ clock cycles. Assuming a relatively low clock frequency of 50 MHz, this would correspond to around 0.5 μs per time step for $N = 10^7$, and thus the total time spent computing the collective momentum for $K = 10^4$ steps would be approximately 5 ms. Hence, we conclude that the computation of the coefficients W_i can be accelerated by approximately a factor of 200 (relative to the implementation using four P100 GPUs) by using computational memory and an FPGA or ASIC-based memory controller.

We can also make a comparative study between the GPU implementation and computational memory with respect to energy to solution. From Supplementary Table 1, we expect the energy to solution for the full kernel to be 148.2 J (The difference between average power and idle power multiplied by the execution time). The energy consumed for just the momentum calculation is 1.68 J. The energy consumption associated with the computational memory unit is difficult to quantify given that we do not have a complete system yet. However, we can make an estimate based on the existing devices and their energy consumption. The energy consumed per device is approximately 580 pJ for one RESET operation and approximately 1.5 pJ for one SET operation. Hence the total energy consumed in the devices for the experiment corresponding to Figure 5 in the manuscript is 58.7 mJ. If we extrapolate this result for the number of processes being $N = 10^7$ and retaining the number of time instances as $K = 10^4$, then the energy consumed is estimated to be approx. 587 mJ. These calculations are based on devices fabricated in the 90 nm technology node. The RESET energy, which dominates the overall energy consumption, will reduce substantially if we consider the state-of-the-art PCM devices fabricated in lower technology nodes. However, this estimate does not consider the overhead associated with read/write circuitry, data converters, control and command circuitry etc. But, even if we assume a substantial

Metric	Momentum only	Full kernel
Integer Instructions	128 B	272 B
Bit-Convert Instructions	102 B	205 B
Control-Flow Instructions	3.2 B	6.4 B
Floating Point Operations (Single Precision Add)	102 B	102 B
Floating Point Operations (Single Precision Mul)	0	**102 B**
Shared Load Transactions	100 k	110 k
Shared Store Transactions	60 k	60 k
Global Load Transactions	800 M	**26 B**
L2 Transactions (Texture Reads)	400 M	800 M
L2 Transactions (Atomic Requests)	0	**204 B**
L2 Throughput (Atomic Requests)	0 GB/s	**788.27 GB/s**
Kernel Execution Time	80 ms	3.9 s
Power (Idle)	35 W	35 W
Power (Avg.)	56 W	73 W

Supplementary Table 1. Profiling of the computational kernel for $K = 10^4$ and $N = 10^7$ on a single P100 GPU.

overhead, the energy consumed for just the momentum calculation is expected to dominate the overall energy consumption, assuming that the momentum is computed using GPU. In this case, we expect an almost two orders of magnitude improvement in the overall energy consumption. If the momentum computation is realized in an FPGA or ASIC-based controller as proposed earlier, one may expect even more substantial improvements.

SUPPLEMENTARY NOTE 7: EMULATION OF COMPUTATIONAL MEMORY IN CMOS

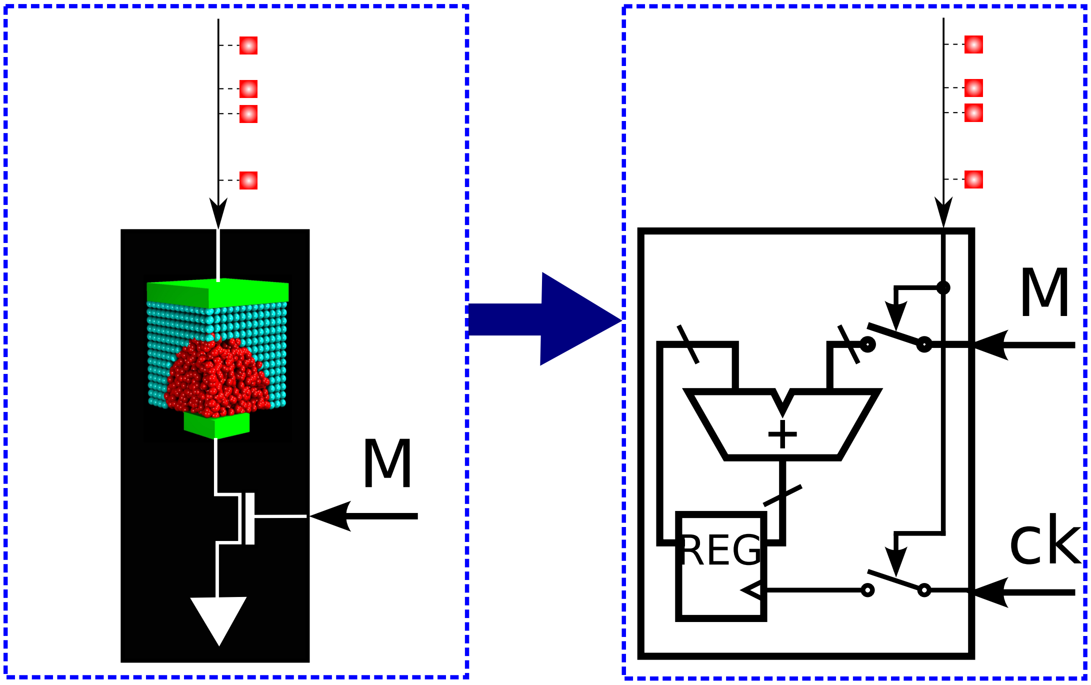

Supplementary Figure 7. Emulation of the accumulation behavior using CMOS technology. One could emulate the accumulative behavior of the PCM using adders and registers. However, such a device is volatile and has a much larger footprint.

One could make an argument for the design of an application-specific chip where the accumulative behavior of PCM is emulated using complementary metal-oxide semiconductor (CMOS) technology using adders and registers (see Supplementary Figure 7). The required resolution of the adder and register is $\log_2(N \times K)$. For 10^6 parallel processes and 4000 time steps, 32 bits are sufficient, but precision could be reduced to save area and power. Comparing at the same technology node, a digital CMOS adder would be on the order of a few 100 transistors, which would require an area that is two orders of magnitude larger than a PCM device with a single access transistor. The much smaller area of a PCM device than that of the CMOS equivalent circuit allows a more than two orders of magnitude higher density on chip. This enables significantly larger problems to be solved in a PCM chip without moving data between the PCM chip and external memory.

Furthermore, the non-volatility of PCM devices enables efficient low-power devices, in which the leakage of CMOS would dominate the dynamic power because of the low utilization rate. We can exploit the enormous storage capacity and non-volatility of PCM devices. The non-volatility makes them particularly attractive for very slow processes. Note that the comparison on current PCM technology discounts the fact that the energy figures corresponding to the computational memory will improve by orders of magnitude as we scale to smaller dimensions[5] and faster programming speeds[6]. We do not foresee such a scaling scenario for CMOS technology[7].

SUPPLEMENTARY NOTE 8: OTHER COMPUTATIONAL PRIMITIVES USING CRYSTALLIZATION DYNAMICS

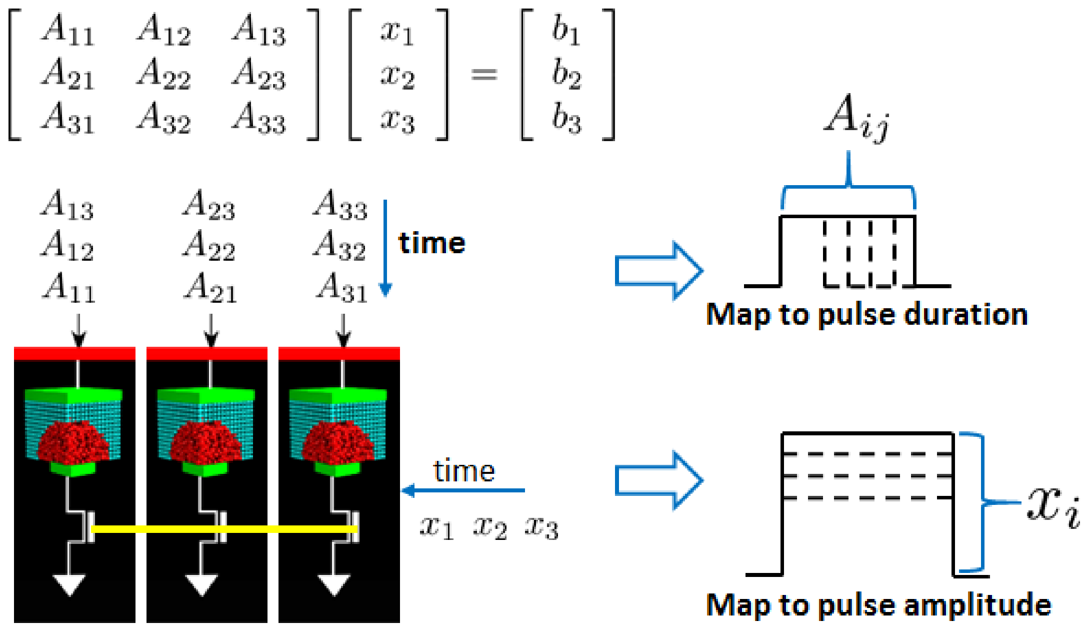

Supplementary Figure 8. **Applications beyond correlation detection.** To multiply a matrix, A, with a vector, x, one PCM device is assigned to each row of A. The elements of A and x are mapped to the pulse characteristics (duration and amplitude, respectively) of the programming pulse. Owing to crystallization dynamics, the result gets stored as the conductance values of the devices.

Temporal correlation detection is one of the computational primitives realizable using crystallization dynamics. Here, we show how we can exploit the crystallization dynamics to perform matrix-vector multiplications (Supplementary Figure 8). Matrix-vector multiplications, such as the one where we would like to multiply a matrix, A, with a vector, x, to obtain the vector, b, arise in a wide range of engineering and scientific applications. As shown in Supplementary Figure 8, one PCM device is assigned to each row of matrix A. Crystallizing pulses of duration proportional to the elements of the row of the matrix are then applied sequentially in time to the corresponding PCM device. The corresponding programming pulse amplitude (programming current) is modulated in accordance with the elements of the vector, x. For example, the first pulse applied to the PCM device corresponding to the first row of the matrix would have an amplitude proportional to x_1 and a duration proportional to A_{11}. Because of the crystallization dynamics, the extent of crystallization and hence the conductance of the device will be proportional to the magnitude of the corresponding element of vector b. The result of the calculation will be “imprinted” as resistance or conductance value of the devices. This is yet another clear illustration of collocated computing and storage. Note that such an approach could scale remarkably well as it would only need N devices for an $N \times N$ matrix.

SUPPLEMENTARY NOTE 9: COMPUTATIONAL MEMORY USING THE DYNAMICS OF STRUCTURAL RELAXATION IN ADDITION TO CRYSTALLIZATION DYNAMICS

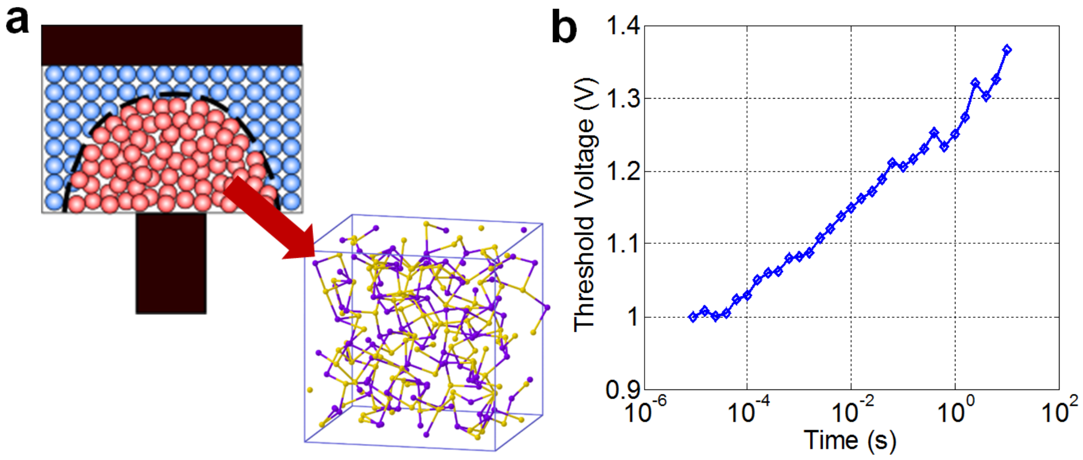

Supplementary Figure 9. **Structural relaxation and its impact on electrical transport.** (**a**) When an amorphous phase is formed by the melt-quench process, the atomic configurations are frozen into an unstable glass state, which relaxes over time to a more stable "ideal" glass state. This is known as structural relaxation and is well captured by a collective relaxation model[11]. (**b**) Experimental data shows that at constant temperature, the structural relaxation manifests itself as a linear increase in the threshold switching voltage, V_{th}, with the log of time.

Phase-change devices exhibit a rich dynamic behavior captured by a feedback interconnection of electrical, thermal and structural dynamics. Besides the crystallization dynamics, we can also exploit other types of dynamics for computational memory. One such dynamic behavior is structural relaxation. When an amorphous phase is formed by the melt-quench process, the atoms are frozen into an unstable glass state (Supplementary Figure 9(**a**)). Subsequently, these atomic configurations relax over time to a more stable "ideal" glass state[8]. The exact nature of this structural relaxation and the nature of the "ideal" glass state are being actively researched[9,10]. We recently showed that the dynamics of structural relaxation can be modelled accurately via a collective relaxation model[11]. The amorphous structure collectively rearranges, whereby every local configuration is changed repeatedly to achieve an overall lower energy state. The relaxation proceeds in a sequence of transitions between neighboring states. The closer to the equilibrium the systems is, the higher is the barrier for subsequent relaxation. If $\Sigma(t) \in [0\ 1]$ denotes an order parameter that indicates the distance of the amorphous state from the ideal glass state at any point in time, t, then $\Sigma(t)$ evolves according to

$$\frac{d\Sigma(t)}{dt} = -\kappa e^{\left(-\frac{E_s}{k_{\text{B}}T(t)}\right)} e^{\left(\frac{\Sigma(t)E_s}{k_{\text{B}}T(t)}\right)}. \tag{14}$$

It can be shown that at constant temperature, $\Sigma(t)$ varies linearly with the log of time.

Experimental measurements such as the one shown in Supplementary Figure 9(**b**) indicate that the threshold-switching field depends on $\Sigma(t)$ as given by

$$E_{\text{th}}(t) = E^0_{\text{th}} + \beta(1 - \Sigma(t)). \tag{15}$$

Hence the threshold switching voltage evolves according to

$$V_{\text{th}}(t) = E_{\text{th}}(t)u_{\text{a}}. \tag{16}$$

Therefore, when programming a PCM device, the applied voltage, V, has to be greater than $V_{\text{th}}(t)$ to pass sufficient current through the PCM device and to induce crystallization. This means that the temperature at the amorphous–crystalline interface, T_{int}, is given by

$$T_{\text{int}} = R_{\text{th}}(u_{\text{a}})\frac{V^2}{R_{\text{ON}}} + T_{\text{amb}}, \text{ if } V > V_{\text{th}}(t) \tag{17}$$

$$= T_{\text{amb}} \text{ If } V \leq V_{\text{th}}(t), \tag{18}$$

where R_{ON} is the high-field ON resistance of the PCM device (largely independent of the amorphous thickness). When the applied voltage, V, is lower than $V_{\text{th}}(t)$, there is no significant Joule heating possible and T_{int} will remain close to T_{amb}. So if we

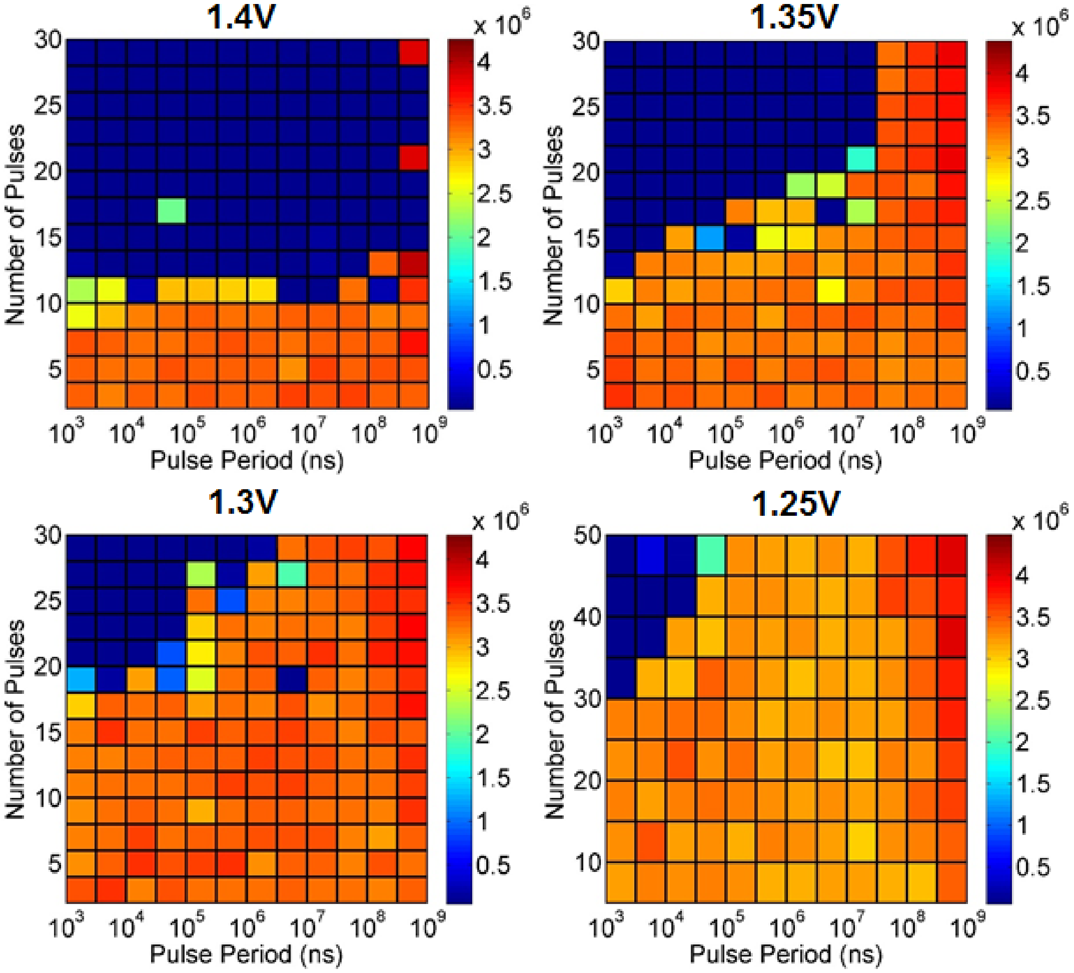

Supplementary Figure 10. **Influence of structural dynamics on the accumulation behavior**. Experimental data showing the dependence of the number of pulses to crystallize on the pulse period. The number of pulses required to reach a threshold conductance level is obtained as a function of the pulse period. When the crystallizing pulse amplitude is large compared with $V_{\text{th}}(t)$ (e.g., 1.4 V), the number of pulses to crystallize is mostly independent of the pulse period. But when the crystallizing pulses have amplitudes comparable to $V_{\text{th}}(t)$, there is a significant dependence on the pulse period. For example when the crystallizing pulse amplitude is 1.25 V, if the pulse period is greater than 10^5 ns, then it is not possible to fully crystallize the phase-change material.

want to exploit the dynamics of structural relaxation in addition to the crystallization dynamics, the key idea is to operate with programming voltages close to $V_{\text{th}}(t)$.

An experimental demonstration of this concept is presented in Supplementary Figure 10. Crystallizing pulses of 50 ns duration are applied to a phase-change device. The number of pulses required to reach a threshold conductance level is obtained. This experiment is repeated by increasing the period of the pulse sequence (or decreasing the time duration between two consecutive pulses). If the voltage of the crystallizing pulse is sufficiently large compared with the threshold switching voltage (e.g., $V =$ 1.4 V), then the number of pulses required for crystallization is almost independent of the pulse period. However, it can be seen that as V becomes smaller, there will be a strong dependence on the pulse period. A larger weighting period between the consecutive application of two pulses could result in substantial structural relaxation such that the applied voltage becomes smaller than the threshold switching voltage. This behavior arising from the dynamics of structural relaxation can be used to discriminate between high-rate and low-rate input processes, such as in rate-coded processes, in addition to the ability to detect temporal correlations.

*IBM, Power, and POWER8 are trademarks of the of International Business Machines Corporation, registered in many jurisdictions worldwide. Other product or service names may be trademarks or service marks of IBM or other companies.

SUPPLEMENTARY REFERENCES

[1] Davis, J. & Goadrich, M. The relationship between Precision–Recall and ROC curves. In *Proceedings of the 23rd International Conference on Machine learning*, 233–240. ACM, (2006).
[2] Aloise, D., Deshpande, A., Hansen, P., & Popat, P. NP-hardness of Euclidean sum-of-squares clustering. *Machine Learning* **75**(2), 245–248 (2009).
[3] Lloyd, S. Least squares quantization in PCM. *IEEE Transactions on Information Theory* **28**(2), 129–137 Mar (1982).
[4] Arthur, D. & Vassilvitskii, S. How slow is the k-means method? In *Proceedings of the Twenty-Second Annual Symposium on Computational Geometry*, SCG '06, 144–153 (ACM, New York, NY, USA, 2006).
[5] Xiong, F., Liao, A. D., Estrada, D., & Pop, E. Low-power switching of phase-change materials with carbon nanotube electrodes. *Science* **332**(6029), 568–570 (2011).
[6] Loke, D., Lee, T., Wang, W., Shi, L., Zhao, R., Yeo, Y., Chong, T., & Elliott, S. Breaking the speed limits of phase-change memory. *Science* **336**(6088), 1566–1569 (2012).
[7] Horowitz, M. Computing's energy problem (and what we can do about it). In *2014 IEEE International Solid-State Circuits Conference Digest of Technical Papers (ISSCC)*, 10–14. IEEE, (2014).
[8] Ielmini, D., Lavizzari, S., Sharma, D., & Lacaita, A. Temperature acceleration of structural relaxation in amorphous $Ge_2Sb_2Te_5$. *Applied Physics Letters* **92**(19), 193511 (2008).
[9] Raty, J. Y., Zhang, W., Luckas, J., Chen, C., Mazzarello, R., Bichara, C., & Wuttig, M. Aging mechanisms in amorphous phase-change materials. *Nature Communications* **6** (2015).
[10] Zipoli, F., Krebs, D., & Curioni, A. Structural origin of resistance drift in amorphous GeTe. *Physical Review B* **93**(11), 115201 (2016).
[11] Sebastian, A., Krebs, D., Le Gallo, M., Pozidis, H.,& Eleftheriou, E. A collective relaxation model for resistance drift in phase change memory cells. In *IEEE International Reliability Physics Symposium (IRPS)*, MY–5. IEEE, (2015).
