# Supplementary material for: Temporal correlation detection using computational phase-change memory
Source: Nat Commun. 2017 Oct 24;8:1115. doi: 10.1038/s41467-017-01481-9 (PMC5653661; doi:10.1038/s41467-017-01481-9)
Supplement: Supplementary file 2 — Description of Additional Supplementary Files [file 41467_2017_1481_MOESM2_ESM.docx]

**Description of Additional Supplementary Files**

File Name: Supplementary Movie 1

Description: A million processes are mapped to the pixels of a 1000 X 1000 pixel black-and-white sketch of Alan Turing. This movie illustrates the turning on and off of the pixels in accordance with the instantaneous binary values of the processes.

File Name: Supplementary Movie 2

Description: This movie illustrates the evolution of the device conductance over time as measured from the million phase-change memory devices. It can be seen that over time the devices corresponding to the correlated processes go to a high conductance state. All the processes have a uniform rate and the correlation coefficient between any two correlated processes is 0.01.
